# Transcriptomic Analysis Reveals Selective Metabolic Adaptation of *Streptococcus suis* to Porcine Blood and Cerebrospinal Fluid

**DOI:** 10.3390/pathogens6010007

**Published:** 2017-02-15

**Authors:** Anna Koczula, Michael Jarek, Christian Visscher, Peter Valentin-Weigand, Ralph Goethe, Jörg Willenborg

**Affiliations:** 1 Center for Infection Medicine, Institute for Microbiology, University of Veterinary Medicine Hannover, Bischofsholer Damm 15, 30173 Hannover, Germany; anna.koczula@tiho-hannover.de (A.K.); peter.valentin@tiho-hannover.de (P.V.-W.); ralph.goethe@tiho-hannover.de (R.G.); 2 Genome Analytics, Helmholtz Centre for Infection Research, Inhoffenstaße 7, 38124 Braunschweig, Germany; michael.jarek@helmholtz-hzi.de; 3 Institute for Animal Nutrition, University of Veterinary Medicine Hannover, Bischofsholer Damm 15, 30173 Hannover, Germany; christian.visscher@tiho-hannover.de

**Keywords:** *Streptococcus suis*, metabolism, amino acid, RNA-seq

## Abstract

*Streptococcus suis* is a zoonotic pathogen that can cause severe pathologies such as septicemia and meningitis in its natural porcine host as well as in humans. Establishment of disease requires not only virulence of the infecting strain but also an appropriate metabolic activity of the pathogen in its host environment. However, it is yet largely unknown how the streptococcal metabolism adapts to the different host niches encountered during infection. Our previous isotopologue profiling studies on *S. suis* grown in porcine blood and cerebrospinal fluid (CSF) revealed conserved activities of central carbon metabolism in both body fluids. On the other hand, they suggested differences in the de novo amino acid biosynthesis. This prompted us to further dissect *S. suis* adaptation to porcine blood and CSF by RNA deep sequencing (RNA-seq). In blood, the majority of differentially expressed genes were associated with transport of alternative carbohydrate sources and the carbohydrate metabolism (pentose phosphate pathway, glycogen metabolism). In CSF, predominantly genes involved in the biosynthesis of branched-chain and aromatic amino acids were differentially expressed. Especially, isoleucine biosynthesis seems to be of major importance for *S. suis* in CSF because several related biosynthetic genes were more highly expressed. In conclusion, our data revealed niche-specific metabolic gene activity which emphasizes a selective adaptation of *S. suis* to host environments.

## 1. Introduction

*Streptococcus suis* is an important facultative pathogen in pigs and is considered a neglected zoonotic pathogen [1,2]. *S. suis* infections can result in severe pathologies such as septicemia, meningitis and endocarditis [1]. Overall, knowledge of the pathogenesis of *S. suis* infection in pigs as well as in humans is scarce. In healthy pigs, *S. suis* is a frequent commensal of the upper respiratory tract. Thus, colonization and interaction of *S. suis* with epithelial cells is proposed to be the first step for invasive infection [3]. After breaching of the epithelial barrier, streptococci enter the bloodstream and disseminate within the host [4,5]. Bacteremia is considered a prerequisite for meningitis as *S. suis* may translocate from the blood to the brain by crossing the blood-brain barrier or the choroid plexus epithelium which is part of the blood-cerebrospinal fluid (CSF) barrier [6]. The contribution of virulence-associated factors such as the capsular polysaccharide, the cytolytic hemolysin (suilysin) and others related to the different steps of infection have been studied in detail in the past [5]. During infection, *S. suis* encounters different host environments with different nutrient availability. However, how *S. suis* adapts its metabolic activity to host environments is largely unknown [7]. Adaptation of bacterial pathogens to changing environments is accompanied by alterations in metabolic gene expression [8,9,10,11,12,13]. Thus, the gene transcriptional levels under infection-related conditions can be used for a better understanding of *S. suis* pathogenicity. 

In a previous study we analyzed the primary carbohydrate metabolism of *S. suis* grown in porcine blood and CSF; both are body fluids encountered in a systemic infection leading to meningitis. Using [U-^13^C_6_]glucose in isotopologue profiling experiments, we were able to analyze de novo amino acid biosynthesis of *S. suis* in porcine blood or CSF. We found that the central carbon metabolic activity was almost similar in both body fluids. However, these studies suggested different activities of the amino acid metabolism in CSF. For instance, the branched-chain amino acid (BCAA) isoleucine seemed to be preferentially synthesized de novo by *S. suis* in CSF but not in blood. BCAA biosynthesis is a tightly transcriptionally regulated metabolic pathway in bacteria [14]. This prompted us to analyze the transcriptomic adaptation of *S. suis* in more detail. For this, the *S. suis* serotype 2 strain 10 was incubated in porcine blood or CSF for six hours according to our previous study [15] and global gene expression was then compared to growth in THB medium by RNA-seq. 

## 2. Results and Discussion

Our experiments revealed 434 and 422 differentially expressed genes in porcine blood and CSF, respectively, when compared to Todd-Hewitt broth (THB) media. In blood, 356 and 78 genes were higher-expressed and lower-expressed, respectively; whereas in CSF we found 206 higher-expressed genes and 216 lower-expressed genes (Tables S2 and S3). Furthermore, in total, 190 differently expressed genes were similar in blood and CSF. Validation of RNA-seq by reverse transcriptase quantitative polymerase chain reaction (RT-qPCR) revealed similar expression profiles for a selected subset of genes with both techniques (Table S4). 

**Figure 1:**
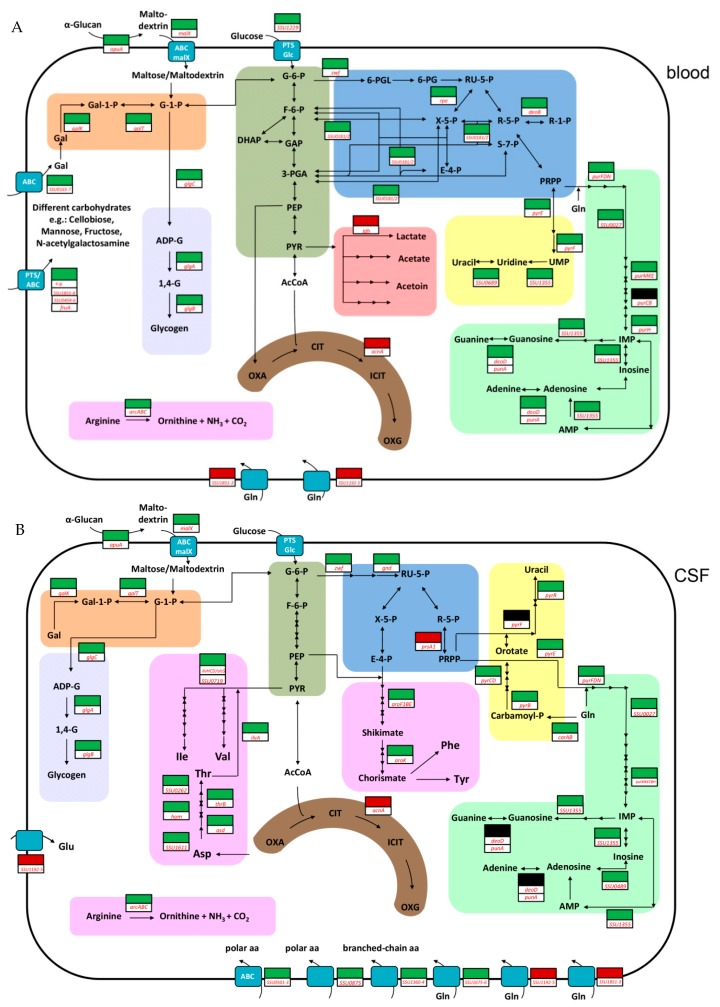
Schematic metabolic model showing important pathways after proliferation of *Streptococcus suis* serotype 2 in porcine blood (**A**) and cerebrospinal fluid (CSF) (**B**). This model is based on the genome annotation of *S. suis* serotype 2 strain P1/7. Important differentially expressed genes found in our study are depicted by colored boxes. Red boxes and green boxes indicate lower- and higher-expressed genes compared to growth in Todd-Hewitt broth (THB), respectively; black boxes indicate that no differential expression was observed. Metabolic pathways are highlighted in different colors and are simplified: dark green, Embden–Meyerhof–Parnas (EMP) pathway (glycolysis); blue, pentose phosphate pathway (PPP); light purple, glycogen metabolism; red, pyruvate metabolism; brown, incomplete tricarboxylic acid (TCA) cycle; orange, Leloir pathway; yellow, pyrimidine metabolism; light green, purine metabolism; pink, amino acid metabolism. For additional information on gene annotations and metabolite abbreviations used, see Supplementary Table S5.

In order to generate energy, imported carbohydrates need to be catabolized by the Embden–Meyerhof–Parnas (EMP) pathway or the pentose-phosphate pathway (PPP). In *S. suis*, the EMP pathway is preferred for the metabolism of glucose [15]. Our RNA-seq data revealed that in blood and in CSF, the expression of EMP pathway genes was unchanged when compared to THB. However, in blood, several genes of the PPP, such as *SSU0181*, *SSU0182*, *zwf* (*SSU1025*), *rpe* (*SSU1785*), and *deoB* (*SSU1269*), showed increased expression (Figure 1A, Table S2). This observation suggests an increasing PPP activity. The higher activity might result from the presence of metabolic intermediates of the EMP such as glucose-6-phosphate, glycerinaldehyde-3-phosphate or fructose-6-phosphate present in *S. suis* grown in blood. As the PPP also delivers metabolites for the synthesis of ribose-5-phosphate, several genes (*purFDNMKEH*, *SSU0027*, *SSU1355* and *deoD*, *punA*) needed for the synthesis of adenine, guanine, and uracil were more highly expressed in porcine blood than in THB medium. In contrast, in CSF only two genes associated with PPP (*zwf*, *gnd*) and purine biosynthesis genes (*purFDNMKECBH*, *SSU0027*, *SSU1355* and *SSU0489*) were more highly expressed. Remarkably, pyrimidine biosynthesis genes (*carAB and pyrBCDER*) were also more highly expressed when compared to THB media, suggesting an enhanced need for uracil with a prolonged time of incubation in CSF. Overall, these data suggested differences in the sugar and nucleotide metabolism when *S. suis* was grown in blood or CSF.

Furthermore, we found that most of the differentially expressed genes in blood (17.5%) encode for proteins involved in the carbohydrate metabolism and transport (G) (Figure S1) which points to blood-specific adaptation. Approximately one-third of the higher-expressed genes in blood are assigned to phosphotransferase systems (PTS) for the uptake of different carbohydrates, such as cellobiose, glucose, sucrose, galactose, *N*-acetylgalactosamine, mannose, fructose, or ascorbate, or ATP-binding-cassette (ABC) transporters such as *msmK* (*SSU1701*), which encodes for an ATPase subunit of ABC transporters and thereby contributes to the utilization of multiple carbohydrates and the host colonization of *S. suis* [16]. These data are in agreement with studies on *Streptococcus agalactiae* in which a higher expression of multiple sugar transport systems was also observed when bacteria were grown in human blood [10]. In addition, we observed an increased expression of genes associated with the Leloir pathway (*galK, galET*) which are involved in the formation of glucose-1-phosphate from galactose. Glucose-1-phosphate may also originate from the degradation of maltodextrins which might be transported by an ABC transporter (*malX*) after enzymatic digestion of more complex α-glucans, such as glycogen by an amylopullulanase (*apuA*) [17,18]. Most likely, glucose-1-phosphate is then introduced into the glycogen biosynthesis pathway encoded by *glgABC* or into the EMP pathway after conversion to glucose-6-phosphate. Overall, the increased expression of genes involved in carbohydrate uptake and conversions might indicate a lack of glucose as a primary sugar source and trigger the uptake of alternative carbohydrates. Indeed, several of these genes have been found to be part of the regulon of the transcriptional regulator catabolite control protein A (CcpA) which is an important regulator of glucose-mediated carbon catabolite repression and activation of metabolic genes in *S. suis* [19,20]. On the other hand, carbohydrate metabolism and transport were also strongly influenced when *S. agalactiae* and *Streptococcus pyogenes* were incubated in human blood for much shorter time periods after which glucose should not yet be exhausted [10,13]. These data suggest that other physiological factors or stress responses mounted by immune cells of the host might also influence the expression profile of streptococci in blood. Further experiments need to be conducted to investigate these hypotheses in more detail.

In contrast to blood, in CSF only a few genes (6.62%) of carbohydrate metabolism and transport (G) (Figure S1) were affected. Accordingly, most of the above-mentioned carbohydrate transporter systems were not more highly expressed when compared to THB, indicating that the utilization of different carbohydrates in CSF seems to be less important when compared to blood. One possible explanation for this might be that in CSF, *S. suis* first has to overcome amino acid starvation. Thus, we found that after growth in CSF, 14.42% of differentially expressed genes were assigned to amino acid metabolism and transport (E) (Figure S1). Respective genes encoding for multiple amino acid transporter systems such as branched-chain amino acids (*SSU1360-SSU1364*), for a polar amino acid ABC transporter (*SSU0501-0503*, *SSU087*5), and for a peptide transport system (*SSU1660-SSU1664*) were increased in expression, indicating the demand of these sources by *S. suis* grown in CSF. Determination of the amino acid concentrations in porcine CSF (Table S6) revealed an approximately 10-fold lower concentration of many amino acids except glutamine in the CSF than in porcine serum, which was in agreement with other studies [21,22]. In line with this observation, the regulation of the operons *SSU1192-1195* (*glnQ4*) and *SSU1851-1853* (*glnQ5*) encoding for two putative glutamine ABC transporter systems was decreased (Figure 1B). Thus, the overall lower concentrations of amino acids (except for glutamine) in the CSF might explain the higher expression of many different amino acid transporter systems after six hours of growth in porcine CSF. Similar observations were made for *S. agalactiae* grown in human amniotic fluid containing low concentrations of free amino acids. Here, genes encoding for amino acid transporter systems were found to be strongly expressed [23,24]. Accordingly, a *Streptococcus pneumoniae* mutant library used in a rabbit meningitis model revealed an important role of genes required for the uptake of amino acids, especially branched-chain and polar amino acids, during experimental meningitis [25]. Notably, we have previously shown that *S. suis* strain 10 is auxotrophic for the amino acids tryptophan, arginine, histidine, glutamine/glutamic acid, and leucine [15]. This might explain the higher expression of the genes encoding for the branched-chain amino acid transporter involved in the import of leucine from CSF. Furthermore, results of our previous isotopologue profiling experiments also suggest that *S. suis* prefers importing tyrosine, phenylalanine, valine, isoleucine and lysine from the environment rather than synthesizing these amino acids [15]. Nevertheless, several genes which are encoding for important precursors for the biosynthesis of isoleucine (*ilvH*/*alaS, ilvC*, *ilvD*, *SSU0719*) and threonine (*SSU1611*, *asd*, *hom*, *thrB*, *SSU0262*, *ilvA*) were highly expressed compared to control and blood conditions (Figure 1B), indicating a higher demand of these amino acids in CSF. This is in agreement with our previous isotopologue profiling studies revealing enhanced isoleucine biosynthesis in CSF but not in blood or chemically defined medium [15]. It has been shown that a functional aromatic amino acid biosynthesis is essential for the virulence of *S. suis* [26]. This might be explained by the low aromatic amino acid concentrations in porcine CSF (Table S6). Thus, in our study several genes (*aroF1*, *aroB, aroE*, *aroK*) of the shikimate pathway, important for aromatic amino acid biosynthesis as shown previously by isotopologue profiling [15], were increased in expression in porcine CSF. 

In conclusion, we observed specific transcriptional profiles of a *S. suis* serotype 2 strain after growth in porcine blood or CSF. In blood, *S. suis* seems to be sufficiently provided with amino acids, whereas a broader spectrum of carbohydrates is used and PPP gene expression is increased. In contrast, in CSF the requirement for amino acids seems to dominate *S. suis* adaptation as genes associated with amino acid transport and biosynthesis, such as those belonging to branched-chain or aromatic amino acid synthesis, were increased in expression. Overall, our results revealed a transcriptional adaptation of *S. suis* to porcine blood and CSF including environment-specific changes of metabolic gene expression in these host environments.

## 3. Materials and Methods

### 3.1. Bacteria and Growth Conditions

Heparinized porcine blood was drawn from healthy pigs, as registered at the Lower Saxonian State Office for Consumer Protection and Food Safety (permit number: 33.9-42502-05-11A137). The collection of blood samples was performed in line with the recommendations of the German Society for Laboratory Animal Science and the German Veterinary Association for Protection of Animals. CSF was taken from clinically healthy pigs and done according to the registered Lower Saxonian State Office for Consumer Protection and Food Safety with the permit number 33.9-42502-04-08/1612. Collected CSF samples were pooled, sterile filtered (0.22 µm Rotilabo^®^ syringe filter), and frozen at −80 °C until use. *S. suis* strain 10 (kindly provided by Hilde Smith, Wageningen UR, The Netherlands) was subcultured on Columbia blood agar plates containing 6% (*v*/*v*) sheep blood (Oxoid). For growth experiments overnight cultures in Todd-Hewitt broth (THB) were adjusted to an OD_600_ of 0.02 in fresh THB on the next day. Bacteria were grown to early-log phase (OD_600_ of 0.2), pelleted, washed in PBS, and resuspended to approx. 10^8^ CFU/mL. Ten mL blood or 2 mL CSF were inoculated with *S. suis* to concentrations of 1 × 10^7^ or 1 × 10^4^ CFU/mL, respectively. Bacteria were incubated under gentle rotation at 37 °C [15]. After 6 h, two volumes of RNAprotect Bacteria Reagent (Qiagen, Venlo, The Netherlands) containing 50 µg/mL chloramphenicol and 5 µg/mL tetracycline were added to the blood and CSF samples. For blood samples, bacteria were immediately separated from blood cells by centrifugation at 400 × *g* for 5 min at 4 °C. The bacteria-containing supernatant was placed in a new collecting tube and centrifuged at 15,550 × *g* for 5 min at 4 °C to pellet the bacteria. Bacteria grown in CSF were pelleted directly from the fluid by centrifugation at 15,550 × *g* for 5 min at 4 °C. As a control, *S. suis* strain 10 was grown in THB medium to an OD_600_ of 0.2, representing the early-log phase of bacterial growth [19], and processed as CSF samples.

### 3.2. RNA Extraction and RNA Deep Sequencing (RNA-seq)

Total RNA from *S. suis* was isolated according to Fulde et al. [27]. Briefly, bacteria grown in porcine blood, CSF and THB medium were resuspended for RNA isolation in 1 mL TRIzol^®^ reagent (ThermoFisher Scientific, Waltham, MA, USA). The cells were then disrupted three times for 45 s at intensity settings 6.5 by using a FastPrep^®^ instrument (Thermosavant, Carlsbad, CA, USA) and cooled on ice. Furthermore, RNA was separated from DNA as well as proteins by chloroform. RNA was precipitated by 2-Propanol and purified by using the RNeasy Mini kit (Qiagen) according to the instructions of the manual that includes a treatment with DNase. RNA quality was verified by gel electrophoresis (2100 Bioanalyser, Agilent Technologies, Santa Clara, CA, USA) and then further processed by the MICROBEnrich™ Kit (ThermoFisher Scientific, Waltham, MA, USA) according to the manufactures recommendation. Sequencing libraries of two independent biological replicates were prepared and sequenced using 50 bp single-ends sequencing on a HiSeq2500 instrument (Illumina, San Diego, CA, USA). In brief, libraries of 300 bp were prepared according the manufacturer’s instructions “TrueSeq RNA Sample Prep Guide” (Illumina). Protocol fragments the RNA, synthesizes first- and second-strand cDNA and ligates adapters to the ends of the cDNA fragments. After PCR enrichment of cDNA fragments, purification with AMPure beads XP and QC on Bioanalyzer/Qbit templates go on the Illumina HiSeq2500 Sequencer system. The fluorescent images were processed to sequences and transformed to FastQ format using the Genome Analyzer Pipeline Analysis software 1.8.2 (Illumina). The sequence output was controlled for general quality features using the fastq-mcf tool of ea-utils [28] and was mapped against the genome sequence of the reference strain *S. suis* P1/7 using BWA version 0.7.5 [29] and SAMtools [30]. Following, all sequences were computed with Rockhopper tool [31]. Genes with a *q*-value ≤ 0.01 were considered as significantly differentially expressed and genes with raw counts in all replicates were included for further analysis.

### 3.3. Reverse Transcriptase Quantitative-PCR (RT-qPCR)

For verification of the RNA-seq data RT-qPCR was performed as previously described [20].

### 3.4. Determination of Amino Acid Concentrations in Porcine Serum and CSF

For the determination of amino acids in porcine CSF and serum samples, 0.25 mL of sulfosalicylic acid was added to 1 mL of CSF or serum, intensively mixed, chilled for 30 min at 4 °C and centrifuged for 10 min at 3000 rpm. The supernatant was mixed 1:1 with sample diluent buffer. Following, amino acids were determined by ion-exchange chromatography (AA analyzer LC 3000, Biotronic, Maintal, Germany) and results evaluated according to established methods [32].

### 3.5. Data Accession Number

Raw data sets are available in the European Nucleotide Archive Repository (PRJEB14724).

## Supplementary Materials

The following are available online at http://www.mdpi.com/2076-0817/6/1/7/s1. Table S1: Bacterial strains and oligonucleotides used in this study. Table S2: Overview of significantly higher- and lower-expressed genes of the *S. suis* wild-type strain 10 in porcine blood as determined by Rockhopper analysis. Table S3: Overview of significantly higher- and lower-expressed genes of the *S. suis* wild-type strain 10 in porcine CSF as determined by Rockhopper analysis. Table S4: Validation of RNA-seq data by RT-qPCR. Table S5: Additional information on gene annotations and metabolite abbreviations used in Figure 1. Table S6: Amino acid concentrations (mg/dL) in porcine serum and CSF. Figure S1: Differentially expressed genes clustered in orthologous groups (COG).
